# Relationship between Socioeconomic Status and Prevalent Prostate Cancer in the South Korea

**DOI:** 10.31557/APJCP.2019.20.10.3137

**Published:** 2019

**Authors:** Hee-Won Hur, So-Yeon Ryu, Jong Park, Seong-Woo Choi

**Affiliations:** 1 *Department of Public Health, Graduate School of Chosun University, *; 2 *Department of Preventive Medicine, Chosun University Medical School, Gwangju, Korea.*

**Keywords:** Prostatic neoplasms, social class, income, educational status

## Abstract

**Background:**

Prostate cancer prevalence recently has increased among male adults in South Korea. But, few study has evaluated the reason. Therefore, we investigated the relationship between socioeconomic status and prevalent prostate cancer.

**Methods:**

This study enrolled 16,215 males aged 40 years and over who took part in the Korean National Health and Nutrition Examination Survey 2007-2016. In addition, we obtained the 2000-2016 age-standardized incidence rate and age-standardized mortality rate of prostate cancer from the Korean Statistical Information Service.

**Results:**

After adjusting for other covariates, prevalent prostate cancer was significantly associated with monthly household income (OR 3.71, 95% confidence interval [CI] 1.48–9.30, for highest vs. lowest) and significantly associated with education level (OR 3.66, 95% CI 1.54–8.70, for ≥ 13 vs. ≤ 6). In the analysis of the age-standardized incidence rate and the age-standardized mortality rate, the age-standardized incidence rate has soared from 2000 to 2011 and then decreased gradually, but the age-standardized mortality rate did not change.

**Conclusion:**

In our results, prevalent prostate cancer increased significantly with socioeconomic status and the increase in prevalent prostate cancer may be attributable to earlier detection rather than to a real increase in prevalence.

## Introduction

Globally, prostate cancer is the most common male cancer (Global Cancer Observatory, 2018) and the number of male patients diagnosed with prostate cancer during the year 2012 was about 1.1 million, and about 70% of prostate cancer cases occurred in developed countries (Global Cancer Observatory, 2018). In the United States, the incidence of prostate cancer peaked in 1992 and subsequently declined, but the prostate cancer incidence is still the highest in American men (Cronin et al., 2018). The new case of prostate cancer in Korea was 3,487 in 2005 (Jung et al., 2009) and increased about 3.4 times to 11,800 in 2016 (Jung et al., 2019).

Age has been known to be the most essential risk factor for prostate cancer; the incidence increases with age after 50 years (Kim, 2004). Also, the incidence of prostate cancer is higher in blacks than in whites, with early onset and malignancy, leading to higher mortality rates (Schwartz et al., 2003). However, none of the risk factors for prostate cancer are clearly known except for age, race, and family history (Platz and Giovannucci, 2006). A westernized lifestyle, obesity, lack of exercise and activity have been reported as risk factors, but the results have been inconsistent (Perez-Cornago et al., 2017; Malik et al., 2018).

Socioeconomic status (SES) is related to health. The lower the SES is, the lower the self-reported health (Borg and Kristensen, 2000) and the increased incidence of illness and mortality (Saydah et al., 2013). However, the relationship between socioeconomic level and cancer incidence and mortality is unclear, and prostate cancer studies have shown varying results. In some studies, the incidence of prostate cancer increased with SES (Cheng et al., 2009), while in other studies it decreased with a higher SES (Baquet et al., 1991), and still other studies found no association between SES and prostate cancer (Williams and Horm, 1977; Mackillop et al., 2000).

However, most of these studies have been performed in Western populations and few studies have been conducted in Korea, where prostate cancer has soared recently. Specifically, few study has evaluated whether prostate cancer in Korea has actually increased because of a specific cause or as a product of early detection. For thyroid cancer, which showed a similar surge, many experts warned that overdiagnosis should be suspected (Choi et al., 2013; Ahn et al., 2014).

Therefore, this study assessed the association of prevalent prostate cancer with SES using the following two data sets: 1) the 2007–2016 Korea National Health and Nutrition Examination Survey (KNHANES), 2) the prostate cancer age-standardized incidence rate (AIR) using cancer registration statistics, and the prostate cancer age-standardized mortality rate (AMR) using cause-of-death statistics released by the Korean Statistical Information Service (KOSIS).

## Materials and Methods

*Subjects*

This study used the 2007–2016 KNHANES data on SES, the prevalent case of prostate cancer, and health behaviors and 2000-2016 KOSIS data on the AIR and AMR of prostate cancer. The details of KNHANES are already demonstrated in previous publication (Kweon et al., 2014). The Korea Centers for Disease Control and Prevention (KCDCP) annually conducts the KNHANES using a sampling design to produce health statistics representative of residents of the Republic of Korea. The KNHANES consisted with the health and nutrition interview and health examination. The health and nutrition interview are conducted by trained interviewers using questionnaires, and the health examinations are performed by trained medical staff. The 2007–2016 surveys included 83,503 participants. After excluded 44,501 women, 19,216 people under 40, and 3,571 people without income, education, or prostate cancer screening data, we analyzed 16,215 men aged 40 years or older.

*Measurements*

Trained investigators interviewed the subjects individually using a questionnaire. A person who answered ‘yes’ to a question about being diagnosed with prostate cancer was defined as a patient with prostate cancer. Monthly household income was classified into quartiles. Education level was divided into ≤ 6, 7–9, 10–12, and ≥ 13 years. BMI was presented by dividing the weight in kilograms by the square of the height in meters. Marriage status was classified as unmarried and married; residence area was divided into urban and rural areas. Current smoking was defined as people who smoked or smoked occasionally, and monthly drinking was defined as having one or more drinking experiences during the previous month. Physical activity was defined as walking for more than thirty minutes at one time and more than 5 times per week. Health checkup in the previous 2 years was defined as a case in which a health checkup had been conducted in the past 2 years; cancer examination in the prior 2 years was defined similarly. Hypertension was defined as taking a hypertensive medicine or blood pressure above 140/90 mmHg; diabetes mellitus was defined as taking a diabetes medicine or using insulin, or fasting blood glucose above 126 mg/dL. Dyslipidemia was defined as taking a dyslipidemic medicine or one of the following four: total cholesterol above 240 mg/dL, triglycerides above 200 mg/dL, low-density lipoprotein cholesterol above 160 mg/dL, and high-density lipoprotein cholesterol below 40 mg/dL. Cardiovascular disease was defined as having been diagnosed with myocardial infarction or angina pectoris or stroke. Other cancer was defined as having been diagnosed with a cancer other than prostate cancer.

*The AIR and AMR of prostate cancer*

The AIR and AMR of prostate cancer in 2000–2016 were analyzed using cancer registration and cause-of-death statistics released on the KOSIS web page (Korean Statistical Information Service, 2018).

*Statistical analysis*

The survey responses were weighted based on a multilevel, multiple, probability sampling design to represent for nationally representative prevalence estimates of the Korean population. The estimates were calculated with consideration for the primary sampling unit, stratification variables, and sampling weights. Data were expressed as estimated percentages (standard errors [SEs]) or mean±standard deviation. The distributions of each variable according to the quartiles of monthly household income and education level were analyzed using the analysis of variance. The associations of prevalent prostate cancer with the quartiles of monthly household income and education level were analyzed using a multivariate logistic regression analysis. Model 1 was adjusted for age, BMI, survey year, marital status, and residence area. Model 2 was additionally adjusted for current smoking, monthly drinking, physical activity, health checkup in the prior 2 years, cancer examination in the prior 2 years, hypertension, diabetes, dyslipidemia, cardiocerebrovascular disease, and other cancer. Model 3 was additionally adjusted for education level or monthly household income. A P-value < 0.05 was considered statistically significant. Statistical analysis was performed using SPSS ver. 18.0.

**Table 1:** General Characteristics of the Subject

Variables	Number	e%(SE) or Mean±SD
Total	16,215	
Prostate cancer patients	69	0.3 (0.0)
Age (year)		55.1±0.1
40-49	4,389	37.6 (0.5)
50-64	6,292	41.8 (0.5)
≥65	5,534	20.6 (0.4)
BMI (kg/m^2^)		24.2±0.0
<18.5	464	2.4 (0.1)
18.5-24.9	9,816	58.7 (0.5)
25.0-29.9	5,480	35.7 (0.4)
≥30	447	3.2 (0.2)
Survey year		
2007	791	4.7 (0.5)
2008	1,779	9.8 (0.6)
2009	2,114	10.4 (0.7)
2010	1,807	10.4 (0.8)
2011	1,791	10.7 (0.8)
2012	1,635	10.5 (0.7)
2013	1,537	10.5 (0.7)
2014	1,442	10.3 (0.7)
2015	1,566	10.8 (0.7)
2016	1,753	12.1 (0.4)
Monthly household income		
Lowest	3,641	17.3 (0.4)
Medium-lowest	4,125	24.9 (0.5)
Medium-highest	4,044	27.4 (0.5)
Highest	4,405	30.4 (0.6)
Education (year)		
≤6	4,131	19.9 (0.4)
7-9	2,572	15.0 (0.4)
10-12	5,033	34.1 (0.5)
≥13	4,479	31.0 (0.6)
Marital status		
Single	1,888	13.6 (0.4)
Married	14,316	86.4 (0.4)
Residential area		
Urban	11,953	77.8 (1.0)
Rural	4,262	22.2 (1.0)
Current smoking	7,768	49.4 (0.5)
Alcohol intake in past month	12,562	80.5 (0.4)
Physical activity^a^	6,796	39.9 (0.5)
Health checkup in the prior 2 years	11,215	68.7 (0.5)
Cancer examination in the prior 2 years	9,375	56.4 (0.5)
Hypertension^b^	7,347	41.9 (0.5)
Diabetes^c^	2,821	16.1 (0.3)
Dyslipidemia^d^	2,695	16.6 (0.3)
Cardiocerebrovascular disease	1,257	5.9 (0.2)
Other cancer	663	3.3 (0.2)

**Table 2:** Characteristics of Subjects by Quartiles of Monthly Household Income

Variables	Monthly household income				P-value
	Lowest	Medium-lowest	Medium-highest	Highest	
Prostate cancer patients	0.4 (0.1)	0.3 (0.1)	0.1 (0.1)	0.4 (0.1)	0.072
Age (year)					<0.001
40-49	14.0 (0.8)	35.6 (1.0)	47.1 (1.0)	45.7 (1.0)	
50-64	31.5 (1.0)	43.4 (0.9)	41.4 (1.0)	47.2 (0.9)	
≥65	54.5 (1.0)	21.0 (0.6)	11.5 (0.5)	7.1 (0.4)	
BMI (kg/m^2^)					<0.001
<18.5	4.9 (0.4)	2.6 (0.3)	1.9 (0.2)	1.0 (0.2)	
18.5-24.9	63.6 (0.9)	59.9 (0.9)	56.1 (0.9)	57.0 (0.9)	
25.0-29.9	28.6 (0.9)	34.3 (0.9)	38.3 (0.9)	38.9 (0.9)	
≥30	2.9 (0.4)	3.3 (0.4)	3.6 (0.4)	3.0 (0.3)	
Survey year					<0.001
2007	6.6 (0.9)	6.0 (0.7)	4.3 (0.6)	2.8 (0.5)	
2008	14.1 (1.2)	11.3 (0.9)	9.9 (0.9)	5.5 (0.7)	
2009	13.3 (1.1)	11.5 (0.8)	10.3 (0.9)	7.7 (0.8)	
2010	9.1 (0.9)	11.3 (1.0)	11.6 (1.0)	9.3 (0.9)	
2011	9.5 (0.9)	10.5 (0.9)	11.0 (1.0)	11.2 (1.0)	
2012	9.3 (1.0)	10.9 (1.1)	11.2 (0.9)	10.2 (1.0)	
2013	9.7 (1.0)	9.9 (0.8)	11.4 (0.9)	10.9 (1.0)	
2014	9.1 (0.9)	9.7 (0.9)	10.6 (0.9)	11.2 (1.1)	
2015	10.1 (1.0)	9.8 (0.9)	9.1 (0.8)	13.7 (1.2)	
2016	9.4 (0.6)	9.2 (0.6)	10.7 (0.6)	17.5 (1.1)	
Education (year)					<0.001
≤6	47.6 (1.0)	23.7 (0.8)	12.2 (0.6)	6.0 (0.4)	
7-9	20.3 (0.8)	19.7 (0.8)	14.2 (0.7)	8.1 (0.5)	
10-12	22.4 (0.8)	38.5 (0.9)	39.1 (1.0)	33.1 (1.0)	
≥13	9.7 (0.6)	18.0 (0.8)	34.5 (0.9)	52.9 (1.1)	
Marital status					<0.001
Single	12.9 (0.7)	11.1 (0.6)	11.8 (0.7)	17.8 (1.1)	
Married	87.1 (0.7)	88.9 (0.6)	88.2 (0.7)	82.2 (1.1)	
Residential area					<0.001
Urban	66.8 (1.6)	74.6 (1.4)	80.6 (1.2)	85.1 (1.2)	
Rural	33.2 (1.6)	25.4 (1.4)	19.4 (1.2)	14.9 (1.2)	
Current smoking	53.0 (1.0)	52.2 (1.0)	50.6 (1.0)	43.7 (1.0)	<0.001
Alcohol intake in past month	68.7 (0.8)	79.6 (0.7)	83.2 (0.7)	86.3 (0.7)	<0.001
Physical activity^a^	43.1 (1.0)	40.4 (0.9)	38.6 (0.9)	38.7 (0.9)	0.004
Health checkup in the prior 2 years	56.6 (1.0)	64.2 (0.9)	70.5 (0.9)	78.6 (0.7)	<0.001
Cancer examination in the prior 2 years	46.6 (0.9)	52.8 (0.9)	56.0 (1.0)	66.1 (0.9)	<0.001
Hypertension^b^	52.2 (1.0)	42.5 (0.9)	39.4 (1.0)	37.2 (0.9)	<0.001
Diabetes^c^	23.9 (0.9)	16.9 (0.7)	13.9 (0.7)	12.7 (0.6)	<0.001
Dyslipidemia^d^	14.9 (0.7)	15.6 (0.7)	15.8 (0.7)	19.3 (0.8)	<0.001
Cardiocerebrovascular disease	12.3 (0.6)	5.3 (0.4)	4.4 (0.4)	3.8 (0.3)	<0.001
Other cancer	5.8 (0.4)	3.4 (0.3)	2.5 (0.3)	2.2 (0.3)	<0.001

All values are given as estimated percentage(standard error); ^a^, Physical activity was indicated as `yes' when the subject walked for more than 30 min at a time and more than five times per week; ^b^, Hypertension was defined as systolic blood pressure ≥ 140 mmHg or diastolic blood pressure ≥ 90 mmHg or taking antihypertension medication; ^c^, Diabetes was defined as fasting serum glucose ≥ 126 mg/dL or taking insulin or oral diabetes medication; ^d^, Dyslipidemia was defined as taking a dyslipidemic medicine or total cholesterol ≥ 240 mg/dL or triglycerides ≥ 200 mg/dL or low-density lipoprotein cholesterol ≥ 160 mg/dL or high-density lipoprotein cholesterol ≤ 40 mg/dL

**Table 3:** Characteristics of Subjects by Education Level

Variables	Education (year)				P-value
	≤6	7-9	10-12	≥13	
Prostate cancer patients	0.5 (0.1)	0.2 (0.1)	0.2 (0.1)	0.4 (0.1)	0.015
Age (year)					<0.001
40-49	7.5 (0.6)	20.1 (1.1)	44.8 (0.9)	57.4 (0.9)	
50-64	43.9 (1.0)	55.8 (1.2)	41.8 (0.9)	33.6 (0.9)	
≥65	48.6 (1.0)	24.1 (0.9)	13.4 (0.5)	8.9 (0.4)	
BMI (kg/m^2^)					<0.001
<18.5	4.6 (0.4)	2.4 (0.3)	1.8 (0.2)	1.6 (0.2)	
18.5-24.9	64.1 (0.9)	60.6 (1.2)	58.1 (0.8)	55.1 (0.9)	
25.0-29.9	29.2 (0.9)	34.2 (1.1)	36.6 (0.8)	39.5 (0.9)	
≥30	2.1 (0.3)	2.9 (0.4)	3.6 (0.3)	3.8 (0.3)	
Survey year					<0.001
2007	5.9 (0.8)	5.4 (0.8)	4.5 (0.6)	3.9 (0.6)	
2008	11.5 (1.0)	11.2 (1.0)	9.3 (0.7)	8.5 (0.8)	
2009	11.9 (1.0)	11.4 (1.0)	9.8 (0.8)	9.5 (0.9)	
2010	11.2 (1.2)	11.5 (1.1)	10.6 (0.9)	9.1 (0.9)	
2011	10.8 (1.1)	11.8 (1.2)	10.8 (0.9)	9.8 (0.9)	
2012	10.6 (1.1)	10.0 (1.0)	11.2 (0.9)	9.8 (1.0)	
2013	9.6 (0.9)	10.3 (1.0)	11.2 (0.9)	10.5 (1.0)	
2014	9.0 (0.9)	9.9 (1.0)	10.1 (0.9)	11.4 (1.0)	
2015	10.1 (1.0)	8.3 (0.9)	11.2 (0.9)	12.0 (1.1)	
2016	9.4 (0.6)	10.3 (0.8)	11.3 (0.6)	15.5 (0.9)	
Monthly household income					<0.001
Lowest	44.1 (1.0)	25.0 (1.0)	12.1 (0.5)	5.8 (0.4)	
Medium-lowest	31.1 (0.9)	34.3 (1.2)	29.4 (0.8)	15.2 (0.7)	
Medium-highest	16.1 (0.7)	24.8 (1.1)	30.1 (0.8)	29.2 (0.8)	
Highest	8.7 (0.6)	15.9 (1.0)	28.4 (0.9)	49.9 (1.0)	
Marital status					<0.001
Single	9.8 (0.6)	11.3 (0.8)	13.7 (0.7)	17.0 (1.0)	
Married	90.2 (0.6)	88.7 (0.8)	86.3 (0.7)	83.0 (1.0)	
Residential area					<0.001
Urban	64.9 (1.6)	72.2 (1.5)	78.4 (1.2)	88.2 (0.9)	
Rural	35.1 (1.6)	27.8 (1.5)	21.6 (1.2)	11.8 (0.9)	
Current smoking	49.5 (1.0)	50.3 (1.2)	52.8 (0.9)	45.2 (0.9)	<0.001
Alcohol intake in past month	71.0 (0.8)	80.3 (0.9)	84.0 (0.6)	82.8 (0.7)	<0.001
Physical activity^a^	40.9 (1.0)	37.7 (1.2)	39.6 (0.8)	40.6 (0.9)	0.152
Health checkup in the prior 2 years	62.2 (1.0)	64.0 (1.2)	67.9 (0.8)	76.0 (0.8)	<0.001
Cancer examination in the prior 2 years	51.5 (1.0)	55.4 (1.2)	54.5 (0.8)	62.0 (0.9)	<0.001
Hypertension^b^	50.7 (1.0)	44.3 (1.1)	41.6 (0.8)	35.5 (0.8)	<0.001
Diabetes^c^	21.4 (0.8)	19.8 (0.9)	15.2 (0.6)	12.1 (0.6)	<0.001
Dyslipidemia^d^	13.9 (0.7)	17.9 (0.9)	16.4 (0.6)	18.0 (0.7)	<0.001
Cardiocerebrovascular disease	10.2 (0.6)	8.7 (0.6)	4.8 (0.3)	3.1 (0.3)	<0.001
Other cancer	5.5 (0.4)	3.5 (0.4)	2.7 (0.3)	2.3 (0.2)	<0.001

All values are given as estimated percentage(standard error); ^a^, Physical activity was indicated as `yes' when the subject walked for more than 30 min at a time and more than five times per week; ^b^, Hypertension was defined as systolic blood pressure ≥ 140 mmHg or diastolic blood pressure ≥ 90 mmHg or taking antihypertension medication; ^c^, Diabetes was defined as fasting serum glucose ≥ 126 mg/dL or taking insulin or oral diabetes medication; ^d^, Dyslipidemia was defined as taking a dyslipidemic medicine or total cholesterol ≥ 240 mg/dL or triglycerides ≥ 200 mg/dL or low-density lipoprotein cholesterol ≥ 160 mg/dL or high-density lipoprotein cholesterol ≤ 40 mg/dL.

**Table 4:** The ORs for Prevalent Prostate Cancer by Quartiles of Monthly Household Income and Education Level

Variables	Model 1^a^	Model 2^b^	Model 3^c^
	OR (95%CI)	OR (95%CI)	OR (95%CI)
Monthly household income			
Lowest	1	1	1
Medium-lowest	1.88 (0.90-3.91)	1.95 (0.93-4.09)	1.87 (0.90-3.87)
Medium-highest	1.47 (0.61-3.53)	1.53 (0.62-3.79)	1.23 (0.48-3.15)
Highest	5.54 (2.63-11.68)	5.32 (2.44-11.61)	3.71 (1.48-9.30)
Education (year)			
≤6	1	1	1
7-9	0.58 (0.17-1.91)	0.67 (0.19-2.35)	0.67 (0.19-2.35)
10-12	1.00 (0.40-2.53)	1.08 (0.42-2.79)	1.07 (0.41-2.78)
≥13	3.44 (1.47-8.03)	3.73 (1.58-8.81)	3.66 (1.54-8.70)

^a^, Adjusted by age, survey year, marital status and residential area; ^b^, Adjusted by Model 1 variables plus BMI, current smoking, alcohol intake in past month, physical activity, health checkup in the prior 2 years, cancer examination in the prior 2 years, hypertension, diabetes, dyslipidemia, cardiocerebrovascular disease and other cancer; ^c^, Adjusted by Model 2 variables plus education level or monthly household income level

**Figure 1:**
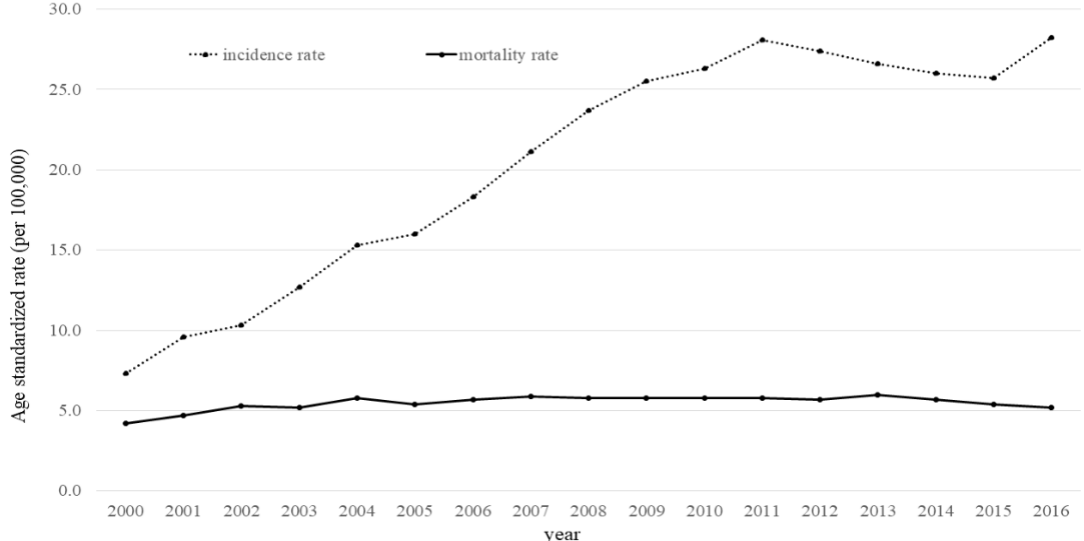
Age-Standardized Incidence and Mortality Rate of Prostate Cancer in 2000-2016

*Ethics statement*

This study was conducted according to the Declaration of Helsinki and all subjects provided informed consent for their data use. The KCDCP ethics committee approved the study protocol (2007-02CON-04-P, 2008-04EXP-01-C, 2009-01CON-03-2C, 2010-02CON-21-C, 2011-02CON-06-C, 2012-01EXP-01-2C, 2013-07CON-03-4C, 2014-12EXP-03-5C, 2015-01-02-6C).

## Results

*General characteristics of the subjects*

Fifty-eight patients were diagnosed with prostate cancer. Their mean age was 55.0 ± 0.1 years, and their mean BMI was 24.2 ± 0.0 kg/m². The lowest to highest quartiles of monthly household income contained 17.3, 24.9, 27.4, and 30.4%, respectively. The education level was ≤ 6 for 19.9%, 7–9 years for 15.0%, 10–12 years for 34.1%, and ≥ 13 years for 31.0% (Table 1). 

*Subject characteristics by quartiles of monthly household income*

Age, BMI, survey year, education level, marital status and smoking differed significantly according to the quartiles of monthly household income. In addition, urban dwellers, monthly household income, health checkup and cancer examination in the prior 2 years, and dyslipidemia significantly increased with increasing monthly household income, while current smoking, hypertension, diabetes, cardiocerebrovascular disease, and other cancer significantly decreased with increasing monthly household income (Table 2). 

*Subject characteristics by education level*

Prevalent case of prostate cancer, age, BMI, survey year, monthly household income, current smoking, monthly drinking, cancer examination in the prior 2 years, and dyslipidemia differed significantly according to the education level. In addition, single person, urban dwellers and health checkup in the prior 2 years increased significantly with education level, while hypertension, diabetes, cardiocerebrovascular disease, and other cancer decreased significantly with increasing education level (Table 3).

*Odds ratios (ORs) for prevalent prostate cancer by quartiles of monthly household income and education level*

After adjusting for age, BMI, survey year, marital status, residence area, current smoking, monthly drinking, physical activity, health checkup and cancer examination in the prior 2 years, hypertension, diabetes, dyslipidemia, cardiocerebrovascular disease, other cancer, and education level (Model 3), prevalent prostate cancer was significantly associated with monthly household income (OR 3.71, 95% confidence interval [CI] 1.48–9.30, for highest vs. lowest). When adjusted for the same variables and monthly household income (Model 3), prevalent prostate cancer was significantly associated with education level (OR 3.66, 95% CI 1.54–8.70, for ≥ 13 vs. ≤ 6) (Table 4).

*The AIR and AMR of prostate cancer*

Figure 1 shows the AIR and AMR of prostate cancer from 2000 to 2016 in South Korea. AIR per 100,000 people increased from 7.3 in 2000 to 28.0 in 2011. It gradually decreased after 2011, reaching 25.5 in 2015 and increased again to 28.2 in 2016. However, the AMR per 100,000 did not change significantly from 4.2 in 2000 to 5.2 in 2016.

## Discussion

This study investigated the relationship between SES and prevalent prostate cancer using 2007–2016 KNHANES data and the trends of the AIR and AMR in prostate cancer from 2000 to 2016 using cancer registration and cause-of-death data. In our results, prevalent prostate cancer increased significantly with household income and education level. Also, the AIR of prostate cancer increased sharply from 2000 to 2011, while there was little change in the AMR between 2000 and 2016. 

In the present study, prevalent prostate cancer increased with SES, as evaluated using monthly household income and education level. In previous studies, the relationship between SES and prostate cancer was inconsistent. An analysis in 65,506 patients diagnosed with any cancer using Korean National Health Insurance cancer registration data showed that the high-income group had a 1.28-fold higher risk of prostate cancer than the low-income group (Kim et al., 2012), which is similar to our results. However, in a study of the relationship between income and prostate cancer among adults living in the US and Canada, the authors presented that prostate cancer increased with income in the United States, while income and prostate cancer were not related in Canada (Mackillop et al., 2000). The reason for this difference is that genetic and cultural factors and the medical systems, especially universal health coverage, differ from country to country (Liu et al., 2001). In addition, the association between SES and prostate cancer seems to be influenced by the introduction of the PSA screening test. In the United States, the incidence of prostate cancer was not associated with SES before the introduction of the PSA test. However, since then, the higher the SES, the higher the incidence of prostate cancer (Liu et al., 2001). Even in study conducted in Finland, where economic inequality is low and public health care is universal, the researchers demonstrated that more educated people underwent more PSA tests and had a higher incidence and lower mortality rate of prostate cancer than less educated people (Kilpeläinen et al., 2016).

Our finding that the prevalent prostate cancer increased with SES means one of the following. First, the risk factors for prostate cancer are increased in the high SES group, which actually increases the incidence and prevalence of prostate cancer. However, as described earlier, the risk factors for prostate cancer are still unclear (Platz and Giovannucci, 2006), and there is currently no evidence that the risk factors affecting only the high SES group have surged in recent decades in Korea. Second, the low SES group underwent less prostate cancer screening, leading to a higher mortality rate, which lowered the prevalence of prostate cancer in the lower SES group, suggesting a higher prevalence of prostate cancer in the high SES group. However, this hypothesis cannot explain our finding that the mortality rate of prostate cancer remained almost unchanged in the years 2000–2016. Finally, it is possible that the high SES group underwent more PSA screening and more prostate cancer was found than in the low SES group. Although the Korean national cancer screening program requires the entire Korean population to receive screening for five cancers (stomach, breast, colorectal, cervical, and liver) (Suh et al., 2016), prostate cancer screening is not included in this essential cancer screening program and additional personal expenses are required to receive prostate cancer screening (Kim et al., 2011). Therefore, the higher the SES, the higher the frequency of PSA testing, the greater the detection rate of prostate cancer, the better access to health care, and eventually the higher survival rate. As a result, the prevalent prostate cancer increases with a higher SES.

Our finding that prevalent prostate cancer increased with SES suggests overdiagnosis for several reasons. First, the incidence increased sharply, but the mortality did not change. This is a typical feature of overdiagnosis (Welch and Black, 2010) as seen in overdiagnosis of thyroid cancer in Korea. In addition, our results demonstrated that the incidence of prostate cancer has increased sharply from 2000 to 2011, and has suddenly decreased since 2012. Interestingly, from 2011, the media began to pay attention to overdiagnosis of thyroid cancer (Korea Times, 2014). Since then, the incidence of thyroid cancer in Korea has decreased significantly (annual percentage change in 1999-2011: 22.4, in 2011-2015: -14.4) (Jung et al., 2018), similar to the incidence of prostate cancer. These results suggest that the social impact of the overdiagnosis of thyroid cancer might similarly affect the incidence of prostate cancer. Second, the result is because of the nature of prostate cancer itself. Prostate cancer occurs frequently in men, but progresses very slowly and has a long survival period, so there is controversy over the effects and necessity of early screening (Etzioni et al., 2002). The autopsy results of people who died from causes other than prostate cancer found that 52% of patients over age 50 and 77% of patients over age 70 had prostate cancer (Hoffman, 2011). Third, as in the developed countries, the increase in prostate cancer in Korea is mainly due to an increase in PSA testing. Although PSA tests are mainly used as early screening for prostate cancer, there is little evidence that PSA tests can reduce prostate cancer mortality (Andriole et al., 2012). In a systematic review, the sensitivity and specificity of the PSA test were only 21% and 91%, respectively, based on a level of 4 ng/mL, suggesting that the PSA test is an incomplete screening tool (Wolf et al., 2010). In a follow-up study of PSA screening and control groups in the Prostate, Lung, Colorectal, and Ovarian (PLCO) cancer screening trial with 76,693 men, the PSA screening group had a higher incidence of prostate cancer, but prostate cancer mortality was not significantly different between the two groups (Andriole et al., 2012). The European Randomized Study of Screening for Prostate Cancer (ERSPC) observed the PSA screening group and control group at nine centers in eight countries for 13 years; the mortality rate of the PSA screening group was significantly lower in only two centers (the Swedish and Dutch centers), and there were no significant differences in the other seven centers (Schröder et al., 2014). 

This study has several limitations. First, it was impossible to clearly present cause-and-effect relationships because a cross-sectional survey was used. Second, prevalent prostate cancer was estimated by referring to questionnaire data rather than medical records. Third, there were no data on tumor size, cancer stage, or histopathology.

In conclusion, prevalent prostate cancer increased significantly with household income and education level and the increase in prevalent prostate cancer in Korea may not be due to an actual increase in prevalence, but to early detection such as PSA screening. 
